# Dietary fermented orange peel and selenium nanoparticles improve antioxidant status, immunity, intestinal integrity, and growth performance in heat-stressed broilers

**DOI:** 10.1016/j.psj.2026.107623

**Published:** 2026-08-15

**Authors:** Haifa Ali Alqhtani, Mohamed Marzok, Ahmed M. Elbaz, Eman Kamel M. Khalfallah, Fatmah Ahmed Safhi, Mohammed Al-Rasheed, Mahmoud HA Mohamed, Mayyadah Abdullah Alkuwayti, Mohamed S. Ahmed, AbdelRahman Y. Abdelhady

**Affiliations:** a Department of Biology, College of Science, Princess Nourah bint Abdulrahman University, P.O. Box 84428, Riyadh, 11671, Saudi Arabia; b Department of Clinical Sciences, College of Veterinary Medicine, King Faisal University, P.O. Box 400, Al-Ahsa, 31982, Saudi Arabia; c Animal and Poultry Nutrition Department, Desert Research Center, Mataria, Cairo, 11753, Egypt; d Biochemistry, Toxicology and Feed Deficiency Department, Animal Health Research Institute, Agricultural Research Center, Dokki, 12616, Giza, Egypt; e Department of Biological Sciences, College of Science, King Faisal University, P.O. Box 400, Al-Ahsa, Saudi Arabia; f Poultry Production Department, Faculty of Agriculture, Ain Shams University, Hadayek Shoubra, Cairo, 11241, Egypt

**Keywords:** Orange peel, Selenium, Fermentation, Growth performance, Heat stress

## Abstract

Heat stress is a major obstacle to the sustainability of poultry production, especially since modern breeds are highly sensitive to stress due to their high metabolic rate and limited ability to dissipate excess heat. This study investigated the effect of dietary supplementation with fermented orange peel and selenium nanoparticles on growth performance, oxidative stability, immune response, gut microbiota, and gene expression in heat-stressed chickens. Three hundred and twenty broiler chicks (Ross 308) were randomly distributed to four treated (8 replication/ group): a group fed a basal diet (control group, CON), and three groups fed a basal diet added with fermented orange peel (50 g/kg, FOP), selenium nanoparticles (0.2 mg/kg, SeNPs), and their mixture (MFOSe), respectively. Dietary MFOSe enhances body weight gain, feed conversion ratio, and carcass weight, while reducing abdominal fat. Serum decreased triglycerides, cholesterol, and uric acid levels, and increased triiodothyronine (T3) levels in broilers fed MFOSe-mixture. Moreover, supplementing MFOSe increases IgA, IgG, interleukin-10 (IL-10), glutathione peroxidase (GPx), catalase (CAT), total antioxidant capacity (T-AOC), and superoxide dismutase enzymes (SOD), whereas interleukin-6 (IL-6) and malondialdehyde (MDA) levels decreased. However, the MFOSe-mixture reduced pathogenic microbial content and upregulated expression of insulin-like Growth Factor 1 (*IGF-1*), mucin 2 (*MUC-2*), sodium-glucose co-transporter-1 (*SGLT-1*), zonula occludens-1 (*ZO-1*), Claudin-1 (*CLDN-1*), and cationic amino acid transporter-1 (*CAT-1*) gene. The study concluded that adding MFOSe-mixture enhanced immunity, antioxidant capacity, intestinal integrity, and growth performance in heat-stressed broilers.

## Introduction

The poultry industry faces numerous challenges that affect its sustainability, among them heat stress (Abdel-Moneim et al., 2021). Heat stress, particularly in regions with high ambient temperatures, is a major obstacle in poultry farming, especially since modern breeds are highly sensitive to heat stress due to their limited capacity to dissipate excess heat, rapid growth, and high metabolic rate (Onagbesan et al., 2023). Heat stress leads to a decrease in feed intake and feed conversion efficiency, along with several physiological changes, including impaired immune function, increased oxidative stress, increased susceptibility to disease, and intestinal damage (Apalowo et al., 2024). This results in reduced nutrient absorption, increased mortality rates, and greater economic losses (Abdel-Moneim et al., 2022). From a sustainability perspective, heat stress exacerbates resource inefficiencies and feed utilization, leading to increased greenhouse gas emissions and nutrient waste, which threatens the long-term sustainability of poultry projects, particularly in hot climates (Attia et al., 2024). To mitigate these heat stress challenges, several strategies have been proposed, including nutritional interventions (Abdel-Moneim et al., 2021). Numerous previous reports have indicated that the use of functional feed additives plays a pivotal role in improving nutrient utilization (Elbaz et al., 2026), thereby reducing greenhouse gas emissions and nitrogen loss (Attia et al., 2024). This is especially true for supplements with antimicrobial, anti-inflammatory, and antioxidant properties, including probiotics, plant extracts, antioxidants, organic acids, oil, and vitamins (Elbaz et al., 2024; Abdel-Moneim et al., 2025). Furthermore, incorporating alternative feed ingredients and industrial agricultural byproducts contributes to the sustainable use of resources and reduces reliance on conventional feeds (Elbaz et al., 2026).

By-products are a form of agricultural waste that offers promising opportunities to enhance the sustainability of the poultry industry (Bist et al., 2024). Using by-products contributes to reducing environmental pollution and promotes a circular economy by recycling nutrients (Kazemi et al., 2025), making them easier to incorporate as alternative feed ingredients for poultry. Among the by-products that generate significant quantities of orange juice, also known as orange pulp, is often treated as waste (Zoidis et al., 2022). This pulp, which accounts for approximately 50% of the weight of fresh oranges, comprises peels, pulp, seeds, and membranes (Abd El Latif et al., 2023). Orange pulp meal (OPM) is highly nutritious due to its high carbohydrate content and bioactive compounds such as flavonoids and phenolic acids, which exhibit antioxidant effects (Zoidis et al., 2022; Abd El Latif et al., 2023). However, it also contains a high percentage of fiber, which is considered a poor feed ingredient in poultry diets (Goliomytis et al., 2025). Therefore, bacterial fermentation of orange pulp can be used to reduce antinutrients and improve its nutritional properties. Fermentation has been shown to be a biotechnological technique that breaks down a wide range of antinutrients, such as complex carbohydrates, using diverse fermenting microorganisms like *Lactobacillus* (Elbaz et al., 2023). Numerous reports have also highlighted the role of bacterial fermentation in improving nutrient availability and producing a fermentation substrate that acts as an antimicrobial and exhibits antioxidant activity, making it a potentially effective method for poultry health (Predescu et al., 2024).

Selenium plays a vital role as an essential element in maintaining physiological homeostasis, particularly through its role in regulating immunity and antioxidant defense systems (Li et al., 2025). It has numerous beneficial effects, including protecting cellular components from oxidative damage (Li et al., 2025). It is a key component of glutathione peroxidases, which neutralize reactive oxygen species and reduce lipid peroxidation (Attia et al., 2024). However, with the development of nanotechnology, selenium nanoparticles have emerged as a promising alternative to traditional selenium sources, proving their effectiveness due to their robust and rapid physical and chemical properties (Ibrahim et al., 2020). In particular, selenium nanoparticles exhibit lower toxicity, higher bioavailability, a larger surface area, greater reactivity, and enhanced biological activity compared to organic and inorganic selenium forms (Li et al., 2025). Due to the aforementioned advantages, incorporating selenium nanoparticles into poultry feed can represent an effective strategy for promoting overall health and growth performance of poultry (Elbaz et al., 2026), especially under stress conditions.

Given the many previous reports highlighting the role of orange peel, selenium, and fermentation in enhancing chickens' performance (Abdel-Moneim et al., 2022; Abd El Latif et al., 2023; Elbaz et al., 2023). We hypothesize that a diet containing a combination of fermented orange peel and selenium nanoparticles may improve oxidative stress, immunity, and gut integrity in heat-stressed chickens. Therefore, we investigated the effects of supplementing fermented orange peel, selenium nanoparticles, and their combination on oxidative stability, immune function, gut microbiota, stress markers, and certain sinusoidal expression associated with gut integrity.

## Methods and materials

### Ethics statement

All animal handling procedures complied with the guidelines of the Institutional Animal Care and Use Research Ethics Committee at the King Faisal University, Saudi Arabia, which approved this study under protocol # KFU-REC-2026-MAY– ETHICS4205.

### Preparing experimental supplements

Nano-selenium was obtained from the Nanotechnology Laboratory at the National Research Centre, Cairo, Egypt. Orange peel (OPM) was purchased from the Juhayna Juice Company, Cairo, Egypt. After receiving the orange peel, it was air-dried at room temperature and then ground. Samples of the dried and ground orange peel were taken for chemical analysis (Table 1). *Lactobacillus acidophilus* (CECT 4529) was obtained from the Microbiology Department, Faculty of Agriculture, Ain Shams University. To begin the fermentation process, the moisture content of the ground orange peel was increased to 30%. Continuous stirring was then used to ensure uniform moisture levels. Next, *L. acidophilus* (1 × 10^9^ CFU/kg) was added to the moist orange peel, with further stirring to ensure the microorganisms were evenly distributed throughout the mixture. The mixture was then placed in a sterile bag (breathing bags with a 1-way breathing valve) prepared for the fermentation process. The fermentation process continued for five days at 32°C. After fermentation, samples of the fermented orange peel (FOP) were taken to count the number of live colonies in colony-forming units (CFUs) per gram of fermented sample. The fermented orange peel was then dried at 35°C for three days before being mixed with experimental feed in two groups: one group alone and the other with nano-selenium. In addition, samples of dried fermented orange peel were taken for the necessary chemical analyses by AOAC methods (2000, Table 1).

**Table 1:** This is a table. Tables should be placed in the main text near to the first time they are cited.

Parameter	OPM	FOP
Dry matter	89.4	90.1
Crude protein	5.61	7.22
Crude Fat	10.4	10.7
Crude Fiber	12.5	9.36

### Preparing experimental supplements

Three hundred and twenty Ross 308 chicks were obtained from Al Wadi Company in Giza, Egypt. The chicks were divided into four experimental groups in a completely randomized design, with 8 replicates per group, each containing 10 chicks. The first group received a basal feed consisting of corn and soybean meal without additives (the control group, CON); however, second, third, and fourth groups received a basal feed supplemented with fermented orange peel (50 g/kg, FOP), selenium nanoparticles (0.2 mg/kg, SeNPs), and a mixture of these (MFOSe), respectively. In accordance with the recommendations of the National Research Council (NRC, 1994), a basic diet was prepared based on the nutritional requirements of Ross 308 chicks as shown in Table 2. From day one to day ten, the chicks were fed crumbled feed, while for the rest of the trial period, they were fed pellet feed. They were also allowed to consume feed and fresh water freely. Humidity and temperature were controlled during the first week. Chicks were received at 32°C for the first two days, then the temperature was gradually reduced to 30°C by the seventh day. Afterward, the chicks were reared under natural climatic conditions, and temperature and humidity were recorded daily between 1:00 and 2:00 PM to assess the temperature and humidity index (THI, Abdel-Moneim et al., 2025). The average temperature was 33.6°C, and the relative humidity was 48.2%, resulting in THI value of between 30.8 and 31.6. The lighting program was one hour of darkness and 23 hours of light starting from the second day, then it was increased to 6 hours of darkness and 18 hours of light.

**Table 2:** Ingredients and calculated chemical compositions of the experimental diets used.

Ingredients	Starter		Grower	
	CON	FOP	CON	FOP
Fermented orange peel	0.00	5.00	0.00	5.00
Yellow corn	52.2	46.3	56.4	49.6
Soybean meal, 44%	32.9	33.1	27.6	28.4
Sunflower meal	3.00	3.00	3.00	3.00
Corn gluten meal	5.00	5.00	5.00	5.00
Di-calcium phosphate	2.05	2.05	2.10	2.10
Calcium Carbonate	1.30	1.30	1.25	1.25
NaCl	0.25	0.25	0.25	0.25
DL –Methionine	0.20	0.20	0.17	0.17
L-lysine	0.00	0.00	0.10	0.10
Soybean oil	2.80	3.50	3.83	4.83
Mineral-Vitamin mixture*	0.30	0.30	0.30	0.30
**Nutrient content**				
Metabolizable energy (kcal/kg)	3000	3000	3100	3100
Crude protein,%	23.0	23.0	21.0	21.0
Crude fiber,%	3.71	3.94	4.18	4.37
Calcium,%	1.06	1.06	1.04	1.04
Phosphorus,%	0.51	0.51	0.50	0.50
Lysine,%	1.09	1.09	1.06	1.06
Methionine,%	0.58	0.58	0.54	0.54

⁎ Choline chloride, 500 mg; selenium, 0.35 mg; iodine, 1.2 mg; zinc, 115 mg; iron, 50 mg; copper, 25 mg; and manganese, 140 mg; vitamin A 10,000 IU; cholecalciferol, 5000 IU; vitamin E, 80 mg; menadione, 8 mg; thiamine, 3.5 mg; riboflavin, 7.5 mg; pyridoxine, 6.75 mg; cobalamin, 50 μg; nicotinic acid, 62.5 mg; pantothenic acid, 25 mg; folic acid, 2 mg; biotin, 200 μg; and vitamin C, 8.5 mg.

### Performance indicators

Live body weight and feed intake were recorded at 21 and 35 days of age, and daily body weight gain (DWG), daily feed intake (DFI), and feed conversion ratio (FCR, g feed/g weight gain) were calculated. On day 35, 8 chickens were randomly selected from each group for slaughter to assess carcass characteristics. The relative weight of abdominal fat, liver, and dressing (%), as well as the relative weight of immune organs (spleen, thymus, and bursa of Fabricius) were calculated. At 35 days of age, eight broiler chickens were separated from each group and placed individually in digesters to begin a four-day digestion trial. To measure nutrient digestibility, fresh fecal samples were collected from the bottom of each bird twice daily (at 8 a.m. and 8 p.m.), and feed intake was recorded. Following the completion of the digestion experiment at the Faculty of Agriculture, Ain Shams University laboratory in Egypt, feed and fecal samples were analyzed to determine dry matter, crude fiber, nitrogen-free extract, crude protein, and ether extract using AOAC methods (2002).

### Serum metabolites

Blood samples were drawn from the jugular vein before slaughter (35 days) to assess lipid metabolism, liver and kidney health, as well as indicators of immunity and oxidation. After blood samples were drawn, they were centrifuged at 4,000 x g for 15 minutes at 4°C to obtain the serum, which was then stored at −20°C until analysis. Triglycerides (TRI), cholesterol (CHO), glucose, total protein, albumin, low-density lipoprotein (LDL), high-density lipoprotein (HDL), alanine aminotransferase (ALT), aspartate aminotransferase (AST), uric acid, and creatinine (CRE) were determined by an automatic biochemical analyzer (Beckman CX-9, Beckman Coulter, Brea, CA), using commercial kits (Bio Diagnostic, Cairo, Egypt). Corticosteroids (COR) and triiodothyronine (T3) levels were measured using commercial kits as stress markers in serum. Serum immunoglobulin concentrations (IgA, IgM, and IgG) and immunocytokines levels of interleukin-6 (IL-6) and interleukin-10 (IL-10) were determined using commercially available ELISA kits (Bethyl Laboratories Inc., Montgomery, TX, and MyBioSource, San Diego, CA, respectively). Serum malondialdehyde (MDA) content, glutathione peroxidase (GPx), catalase (CAT), total antioxidant capacity (T-AOC), and superoxide dismutase enzymes (SOD) levels were determined using commercial assay kits (Nanjing Jincheng Bioengineering Institute, Nanjing, China) according to the manufacturer's instructions.

### Intestinal integrity

During slaughter, the contents of the cecum (1 gram was taken from each broiler, 8 chickens/group) were collected in sterile bags for microbial estimation using the pour-in-plates method. The samples were diluted 1:10 in 9 ml of Ringer's solution, and then 1 ml of the diluted solution was spread onto the following agar media: VRBD agar, SIA agar, deMan agar, and MRS agar to enumerate coliforms, *Clostridium perfringens, Escherichia coli*, and *Lactobacillus* counts, respectively, following the procedures described by Abdel-Moneim et al. (2025). Additionally, pH level measurement using a pH meter (Basic 20, Crison, Spain): Samples of the contents of intestine digesta were collected and placed in clean centrifuge tubes containing deionized distilled water after thorough homogenization, according to Alqhtani et al. (2026b). Furthermore, the concentrations of short-chain fatty acids (SCFA), primarily acetate, propionate, and butyrate, were determined in samples of cecal contents immediately after withdrawal. Briefly, samples were homogenized, centrifuged, and the supernatant was acidified and filtered before analysis. This was done following the procedures described by Shehata et al. (2022) using a gas chromatograph coupled with a mass spectrometer (Agilent 5975C, Agilent Technologies, Wilmington, DE).

### Intestinal health-associated genes

Total RNA was extracted from broiler intestinal tissue using TRIZOL reagent, according to the manufacturer's instructions (RNAiso Plus; TaKaRa Biotechnology, Dalian, China). Using a UV-Vis nanodrop spectrometer (Q5000, Quawell Technology, San Jose, CA), the concentration and purity of the extracted RNA were assessed. Complementary cDNA was synthesized for each sample according to the manufacturer's instructions (SensiFast™ cDNA Synthesis Kit, Bioline, Product No. Bio-65053). Real-time PCR was performed to estimate the relative mRNA expression levels of intestinal health-associated genes, including insulin-like Growth Factor 1 (IGF-1), mucin 2 (MUC-2), sodium-glucose co-transporter-1 (SGLT-1), zonula occludens-1 (ZO-1), Claudin-1 (CLDN-1), and cationic amino acid transporter-1 (CAT-1). Description of the genes and corresponding primers is as follows: IGF-1 forward: 5′-ATAGAGCCTGCGCAATGGAA-3′, reverse: 5′- ACACAGTGTGCATCTTCACC-3′ (NM_001004384.3), CAT-1 forward: 5′-AGGCTTCCCATCTCACCTCA-3′, reverse: 5′-TCTTCACATGCCATCCAGGT-3′ (NM_001398060.1), ZO-1 forward: 5′CAGCGAAAGAAGGATTAGAGGAA-3′, reverse: 5′-GGAGATCAAGCAAAAAAAGGACA-3′ (XM_015278981.1), MUC2 forward: 5′-CATTCAACGAGGAGAGCTGC-3′, reverse: 5′-TTCCTTGCAGCAGGAACAAC-3′ (JX284122.1), SGLT-1 forward: 5′-AAGGTTTGCACGAGCCAGAT-3′, reverse: 5′-TGTGGACACCAAATGCTTCA-3′ (NM_001293240.2), CLDN-1 forward: 5′- CTTCGTCATGCTCATCGCCT-3′, reverse: 5′-TGATCAGCAGTGCAATTCCT-3′ (NM_001013611.2), and GAPDH gene (NM_204305.2, housekeeping gene) were formed as follows: 5′-AGTCAACGGATTTGGCCGTA-3′, reverse: 5′-ACAGTGCCCTTGAAGTGTCC-3′, with the thermocycler protocol. The relative gene expression levels were quantified using the comparative 2−∆∆Ct method (Livak and Schmittgen, 2001) with the glyceraldehyde-3-phosphate dehydrogenase (GAPDH) gene as a reference for normalization.

### Statistical analysis

All data were analyzed using one-way analysis of variance (ANOVA) with SPSS version 17.0 software, followed by Tukey’s post hoc multiple range test. Gene expression was analyzed using GraphPad Prism version 9.0 (GraphPad Software, San Diego, CA). Normality was also verified before statistical analysis using the Shapiro-Wilk-Levine tests. The data were presented as means ± SEM. Differences among group means were considered statistically significant at *p* < 0.05.

## Results

### Performance and carcass trait

Regarding growth performance, DWG tended to increase in chickens fed FOP and MFOSe compared to SeNPs and control groups (*p* < 0.001), during the period 1–21 days (Table 3). Furthermore, FCR improved in chickens fed FOP and MFOSe compared to SeNPs and control groups (*p* = 0.011), even though the DFI remained unchanged during the same period (*p* > 0.05). Additionally, DWG increased in chickens fed SeNPs, FOP, and MFOSe compared to the control group (*p* < 0.001), during the period 22–35 days. More-over, FCR improved in chickens fed SeNPs, FOP, and MFOSe compared to the control group (*p* < 0.001); however, DFI was higher in chickens fed MFOSe compared to the other groups during the same period (*p* < 0.05). Additionally, DWG increased in chickens fed SeNPs, FOP, and MFOSe compared to the control group (*p* < 0.001), during the period 1 –35 days. Moreover, FCR improved in chickens fed SeNPs, FOP, and MFOSe compared to the control group (*p* < 0.001); however, DFI remained unchanged during the same period (*p* > 0.05). One of the most significant observations is the highest DWG in the group that received MFOSe, while the best FCR is in the group that received MFOSe and FOP during the overall period. Dressing increased in chickens that received SeNPs, FOP, and MFOSe supplements (*p* = 0.001), while abdominal fat decreased in chickens that received FOP and MFOSe supplements compared to the SeNPs and control group. However, relative liver weight was not affected by the experimental treatments (*p* > 0.05).

**Table 3:** Impact of adding fermented orange peel and nano-selenium on growth performance and carcass traits in heat-stressed chickens.

Parameter	CON	FOP	SeNPs	MFOSe	SEM	p-value
1–21 days						
DWG, g	41.59^b^	44.38^ab^	42.09^b^	45.48^a^	0.181	0.001
DFI, g	46.86	48.37	48.41	48.20	0.307	0.103
FCR, g:g	1.18^a^	1.09^b^	1.15^a^	1.06^b^	0.006	0.011
22–35 days						
DWG, g	66.21^d^	71.57^b^	69.21^c^	79.14^a^	0.213	< 0.001
DFI, g	151.9^b^	152.6^b^	149.8^b^	163.4^a^	2.740	0.036
FCR, g:g	2.29^a^	2.13^b^	2.16^b^	2.05^c^	0.031	< 0.001
1–35 days						
DWG, g	50.31^d^	55.26^b^	52.94^c^	58.94^a^	0.137	< 0.001
DFI, g	88.91	90.06	88.93	94.31	1.861	0.067
FCR, g:g	1.76^a^	1.63^c^	1.68^b^	1.60^c^	0.009	< 0.001
**Carcass traits**						
Dressing, %	69.03^c^	71.6^a^	70.4^b^	71.9^a^	1.003	0.001
Liver, %	1.84	1.78	1.80	1.76	0.265	0.223
Abdominal fat, %	0.72^a^	0.53^b^	0.68^a^	0.51^b^	0.082	0.001

CON: chickens fed a basic diet without experimental additives, FOP: chickens fed a basic diet with fermented orange peel, SeNPs: chickens fed a basic diet with selenium nanoparticles, MFOSe: chickens fed a basic diet with fermented orange peel and selenium nanoparticles mixture. DWG: body weight gain, DFI: daily feed intake, FCR: feed conversion ratio.

### Nutrient digestibility

The experimental supplementation had a positive effect on nutrient digestibility (Table 4) via increasing the crude protein digestibility (*p* < 0.001) in chickens receiving SeNPs, FOP, and MFOSe compared to the control group. However, the digestibility of dry matter (*p* = 0.001) and crude fiber (*p* = 0.003) was increased in chickens receiving FOP and MFOSe compared to the SeNPs and control groups. Nevertheless, the ether extract and nitrogen-free extract digestibility were unaffected by the experimental treatments (*p* > 0.05).

**Table 4:** Impact of adding fermented orange peel and nano-selenium on nutrient digestibility in heat-stressed chickens at 35 d.

Parameter	CON	FOP	SeNPs	MFOSe	SEM	p-value
Dry matter, %	70.41^b^	73.52^a^	71.07^b^	74.11^a^	1.335	0.001
Crude protein, %	68.25^c^	71.80^a^	70.33^b^	72.61^a^	0.574	< 0.001
Crude fiber, %	58.57^b^	64.51^a^	59.20^b^	65.02^a^	0.095	0.003
Ether extract, %	81.32	82.26	81.04	82.19	0.821	0.106
NFE, %	64.35	64.80	64.28	65.04	1.052	0.218

CON: chickens fed a basic diet without experimental additives, FOP: chickens fed a basic diet with fermented orange peel, SeNPs: chickens fed a basic diet with selenium nanoparticles, MFOSe: chickens fed a basic diet with fermented orange peel and selenium nanoparticles mixture. NFE: nitrogen-free extract.

### Biochemical indices

To detect biochemical indicators, the effect of experimental supplements on lipid profile, stress index, and liver and kidney functions in heat-stressed chickens was evaluated as shown in Table 5. Compared to the control group, triglyceride (*p* = 0.012) and cholesterol (*p* = 0.007) levels tended to be lower in the groups supplemented with FOP and MFOSe compared to the SeNPs and control groups. Similarly, LDL-cholesterol levels decreased (*p* < 0.001), and HDL-cholesterol levels increased (*p* = 0.001) in the groups supplemented with FOP, MFOSe, and SeNPs compared to the control group. However, uric acid levels decreased (*p* = 0.002) in the groups supplemented with FOP and MFOSe, while creatinine levels increased (*p* < 0.001) in the groups supplemented with FOP, MFOSe, and SeNPs compared to the control group. Comparably, AST levels decreased (*p* = 0.001) in the groups supplemented with FOP, MFOSe, and SeNPs compared to the control group. Nevertheless, the T. protein, albumin, and ALT levels were unaffected by the experimental treatments (*p* > 0.05). Regarding stress indicators, corticosteroid levels decreased, and T3 and glucose levels increased (*p* < 0.001) in the groups supplemented with FOP, MFOSe, and SeNPs compared to the control group.

**Table 5:** Impact of adding fermented orange peel and nano-selenium on serum metabolites in heat-stressed chickens at 35 d.

Parameter	CON	FOP	SeNPs	MFOSe	SEM	p-value
**Lipid profile**						
TRI, mg/dL	246^a^	211^b^	224^ab^	203^b^	0.851	0.012
CHO, mg/dL	216^a^	187^b^	208^a^	181^b^	0.266	0.007
HDL, mg/dL	42.5^c^	48.6^a^	45.3^b^	49.4^a^	0.092	0.001
LDL, mg/dL	121^a^	107^b^	101^b^	92.6^c^	0.115	< 0.001
**Liver and kidney functions**						
T. protein, mg/dL	3.27	3.34	3.21	3.31	1.024	0.171
Albumin, mg/dL	1.39	1.41	1.40	1.38	0.731	0.206
Uric acid, mg/dL	6.51^a^	6.28^b^	6.49^a^	6.13^b^	0.084	0.002
CRE, mg/dL	0.47^c^	0.61^ab^	0.53^b^	0.68^a^	0.108	< 0.001
AST, U/L	67.4^a^	56.7^b^	60.8^b^	55.4^b^	0.037	0.001
ALT, U/L	172	168	177	163	0.058	0.114
**Stress index**						
Glucose, mg/dL	213^b^	251^a^	243^a^	256^a^	0.030	0.001
T3, ng/L	1.19^c^	1.63^ab^	1.46^b^	1.78^a^	0.109	< 0.001
COR, nmol/L	41.5^a^	37.2^b^	38.3^b^	37.6^b^	0.075	0.010

CON: chickens fed a basic diet without experimental additives, FOP: chickens fed a basic diet with fermented orange peel, SeNPs: chickens fed a basic diet with selenium nanoparticles, MFOSe: chickens fed a basic diet with fermented orange peel and selenium nanoparticles mixture. TRI: Triglycerides, CHO: Cholesterol, T. protein: Total protein, CRE: Creatinine, T3: Triiodothyronine, COR: Corticosteroids, LDL: low-density lipoprotein, HDL: high-density lipoprotein, ALT: alanine aminotransferase, AST: aspartate aminotransferase.

### Immune response

Table 6 shows the effect of fermented orange peel and nano-selenium supplements on the immune response in heat-stressed chickens. The relative weight of the spleen in-creased (*p* < 0.001) in the groups receiving FOP and MFOSe supplementation. Similarly, the relative weight of the bursa of Fabricius increased (*p* = 0.002) in the groups receiving FOP, MFOSe, and SeNPs supplementation compared to the control group; however, the relative weight of the thymus was not affected by the experimental supplementation (*p* > 0.05). Additionally, IgA and IgG levels increased (p ˂ 0.001) in the groups receiving FOP, MFOSe, and SeNPs supplements compared to the control group; however, IgM levels were not affected by the experimental supplements (*p* > 0.05). Besides, IL-10 levels increased (*p* ˂ 0.001), and IL-6 levels decreased (*p* = 0.015) in the groups receiving FOP, MFOSe, and SeNPs supplements compared to the control group.

**Table 6:** Impact of adding fermented orange peel and nano-selenium on immune functions in heat-stressed chickens at 35 d.

Parameter	CON	FOP	SeNPs	MFOSe	SEM	p-value
**Immune organs**						
Spleen, %	0.108^b^	0.152^a^	0.122^b^	0.147^a^	0.235	< 0.001
Thymus, %	0.125	0.119	0.131	0.128	0.311	0.413
BF, %	0.132^b^	0.224^a^	0.195^a^	0.216^a^	0.107	0.002
**Immunoglobulin**						
IgA, mg/dL	94.8^c^	116^b^	127^a^	135^a^	0.648	0.001
IgM, mg/dL	67.2	69.5	68.3	68.7	1.003	0.067
IgG, mg/dL	321^d^	394^b^	366^c^	427^a^	0.244	< 0.001
**Immunocytokines**						
IL-6, pg/mL	71.3^a^	58.6^b^	57.4^b^	57.9^b^	0.081	0.015
IL-10, pg/mL	45.4^c^	63.3^b^	69.6^a^	68.5^a^	0.126	< 0.001

CON: chickens fed a basic diet without experimental additives, FOP: chickens fed a basic diet with fermented orange peel, SeNPs: chickens fed a basic diet with selenium nanoparticles, MFOSe: chickens fed a basic diet with fermented orange peel and selenium nanoparticles mixture. BF: bursa of Fabricius, IL-10: interleukin-10, IL-6: interleukin-6.

### Redox State

Adding fermented orange peel and nano-selenium enhanced the redox state in heat-stressed chickens, as shown in Table 7. Compared to the control group, the blood levels of SOD, T-AOC, GPx, and CAT increased (*p* ˂ 0.001, *p* = 0.013, *p* = 0.001, and *p* = 0.001, respectively) in the chickens that received FOP, SeNPs, and MFOSe supplements. Meanwhile, the highest SOD levels were observed in the SeNPs and MFOSe groups; the highest GPx and CAT levels were observed in the MFOSe group. In contrast, MDA blood levels were lower (*p* ˂ 0.001) in the chickens that received FOP, SeNPs, and MFOSe supplements compared to the control group, while the lowest level was in the MFOSe group.

**Table 7:** Impact of adding fermented orange peel and nano-selenium on redox state in heat-stressed chickens at 35 d.

Parameter	CON	FOP	SeNPs	MFOSe	SEM	p-value
SOD, U/mL	29.4^c^	36.2^b^	41.8^a^	42.3^a^	0.015	< 0.001
T-AOC, U/mL	6.57^b^	8.24^a^	8.51^a^	9.11^a^	0.067	0.013
GPx, U/mL	21.8^c^	23.8^b^	25.2^ab^	26.4^a^	0.009	0.001
CAT, U/mL	34.2^c^	37.6^b^	38.2^b^	40.5^a^	0.051	0.001
MDA, nmol/ ml	2.51^a^	1.64^c^	1.95^b^	1.26^d^	0.004	< 0.001

CON: chickens fed a basic diet without experimental additives, FOP: chickens fed a basic diet with fermented orange peel, SeNPs: chickens fed a basic diet with selenium nanoparticles, MFOSe: chickens fed a basic diet with fermented orange peel and selenium nanoparticles mixture. MDA: malondialdehyde, GPx: glutathione peroxidase, CAT: catalase, T-AOC: total antioxidant capacity, SOD: superoxide dismutase enzymes.

### Gut health

Table 8 shows the effect of experimental supplements on gut health during heat stress. The pH value decreased (*p* = 0.001) in chickens supplemented with FOP and MFOSe compared to the control and SeNPs groups. Similarly, the concentrations of acetic acid, butyric acid, and propionic acid decreased (*p* = 0.020, *p* = 0.046, and p ˂ 0.015, respectively) in chickens supplemented with FOP and MFOSe compared to the control and SeNPs groups. Regarding microbial changes, the coliform count tended to decrease (*p* = 0.021) in the groups supplemented with FOP, SeNPs, and MFOSe compared to the control group. Besides, C. perfringens decreased (*p* = 0.010), while Lactobacillus counts increased (p ˂ 0.001) in the groups supplemented with FOP and MFOSe compared to the control and SeNPs groups. Additionally, E. coli count decreased (*p* = 0.003) in the groups supplemented with FOP, MFOSe, and SeNPs compared to the control group.

**Table 8:** Impact of adding fermented orange peel and nano-selenium on cecal microflora count (log₁₀ CFU/g), pH value, and SCFA levels in heat-stressed chickens at 35 d.

Parameter	CON	FOP	SeNPs	MFOSe	SEM	p-value
**pH and SCFA levels**						
pH	6.31^a^	6.12^b^	6.29^a^	6.08^b^	0.534	0.001
Acetic acid	3.75^b^	4.24^a^	3.81^b^	4.19^a^	0.085	0.020
Butyric acid	0.54^b^	0.71^a^	0.51^b^	0.76^a^	0.122	0.046
Propionic acid	0.32^b^	0.45^a^	0.30^b^	0.48^a^	0.301	0.015
**Cecal microbiota**						
Coliforms	7.41^a^	7.19^b^	7.26^ab^	7.15^b^	2.006	0.021
*C. perfringens*	1.58^a^	0.76^b^	1.47^a^	0.80^b^	0.674	0.010
*E. coli*	4.73^a^	1.88^c^	3.65^b^	1.92^c^	0.318	0.003
*Lactobacillus*	5.06^c^	7.05^a^	5.82^b^	7.23^a^	0.525	< 0.001

CON: chickens fed a basic diet without experimental additives, FOP: chickens fed a basic diet with fermented orange peel, SeNPs: chickens fed a basic diet with selenium nanoparticles, MFOSe: chickens fed a basic diet with fermented orange peel and selenium nanoparticles mixture. SCFA: short-chain fatty acids, *C. perfringens: Clostridium perfringens, E. coli: Escherichia coli*.

### Genes

**Fig. 1:**
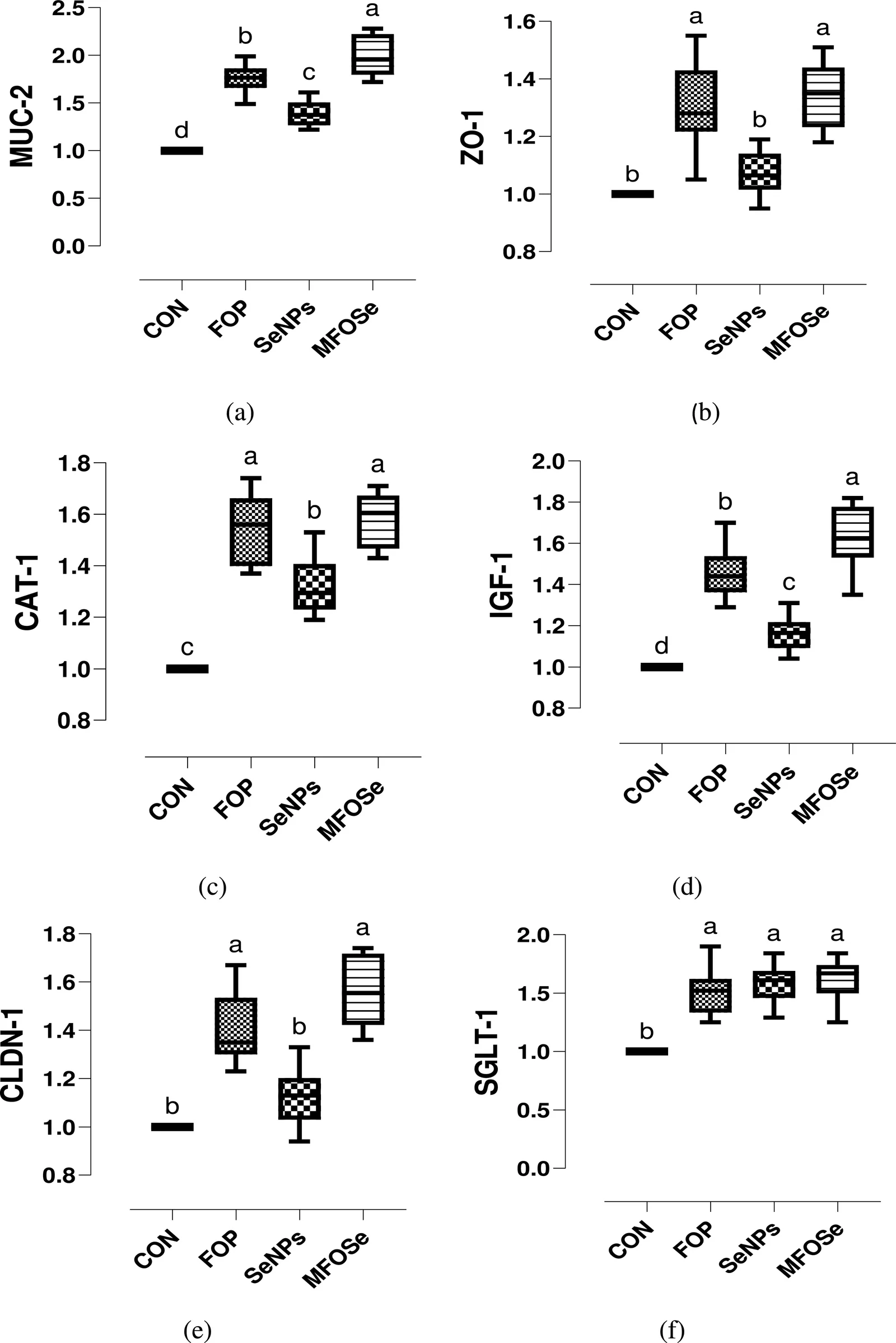
Impact of adding fermented orange peel and nano-selenium on mucin 2 (MUC-2, a), zonula occludens-1 (ZO-1, b), cationic amino acid transporter-1 (CAT-1, c), insulin-like Growth Factor 1 (IGF-1, d), Claudin-1 (CLDN-1, e), and sodium-glucose co-transporter-1 (SGLT-1, f) in broiler chickens at 35 days. CON: chickens fed a basic diet without experimental additives, FOP: chickens fed a basic diet with fermented orange peel, SeNPs: chickens fed a basic diet with selenium nanoparticles, MFOSe: chickens fed a basic diet with fermented orange peel and selenium nanoparticles mixture. Significant differences between the groups are indicated by different letters. The data are displayed as the mean± SD.

The data in Fig. 1 demonstrate the positive effect of fermented orange peel with selenium nanoparticles supplementation on modulating gene expression associated with growth and intestinal health. The upregulation of MUC-2, CLDN-1, SGLT-1, and CAT-1 genes was increased (p ˂ 0.001, *p* = 0.011, *p* = 0.030, and *p* = 0.002, respectively) in chickens receiving SeNPs, FOP, and MFOSe compared to the control group (Fig. a, b, c, and d). Similarly, the upregulation of IGF-1 and ZO-1 genes was increased (p ˂ 0.001 and *p* = 0.010) in chickens receiving FOP and MFOSe compared to SeNPs and control groups (Fig. e and f).

## Discussion

The effects of heat stress are not limited to reduced feed intake and growth; they extend to a range of complex disorders, including oxidative, metabolic, immune, intestinal, and molecular disturbances, leading to decreased performance and health in broiler chickens (Abdel-Moneim et al., 2021), as confirmed by the findings of this study. Therefore, feed additives are used as effective strategies to mitigate these effects and improve the birds' heat tolerance, which include antioxidants, amino acids, probiotics, vegetable oils, and organic minerals (Abdel-Moneim et al., 2024; Elbaz et al., 2024). In the study, incorporating fermented orange peel and nano-selenium into chicken diets had an effective impact on reducing the negative effects of stress through their combined antioxidant, gut health-enhancing, and immune-boosting effects.

Growth performance was improved in broilers that received a mixture of fermented orange peel and nano-selenium during heat stress, in this study. This improvement may be attributed to the dietary supplement's role in enhancing antioxidant capacity, immune function, and gut health (Abd El Latif et al., 2023; Elbaz et al., 2026). Orange peel is rich in phenolic compounds, tannins, and flavonoids, which possess potent antioxidant and antimicrobial properties (Goliomytis et al., 2025) that mitigate the damage of heat stress and thus increase nutrient availability and growth rate. Furthermore, the fermentation process enhances the nutritional and functional value of orange peel by reducing anti-nutritional compounds such as complex tannins, producing beneficial organic acids and enzymes, increasing the bioavailability of bioactive compounds, and improving gut microbiome balance, digestion, and nutrient absorption (Zhu et al., 2023; Predescu et al., 2024), thereby enhancing nutrient availability. Additionally, selenium is an essential component in the formation of the antioxidant enzyme glutathione peroxidase, thus enhancing antioxidant defenses, reducing ROS-induced cell damage, improving immune system efficiency, and supporting anti-inflammatory cytokine activity (Abdel-Moneim et al., 2022), thereby enhancing gut health. Consistent with our findings, several previous reports have indicated the positive effects of orange peel, fermented feed, or nano-selenium supplementation on enhancing growth performance (Hussein et al., 2022; Zhu et al., 2023; Elkhateeb et al., 2024). These results suggest that adding a mixture of fermented orange peel and nano-selenium may enhance antioxidant capacity, improve intestinal health, and regulate immune function, positively impacting growth performance in heat-stressed broilers.

Heat stress leads to decreased carcass weight and increased abdominal fat deposition in chickens due to metabolic disturbances (Abdel-Moneim et al., 2021). However, in the current study, the addition of a mixture of fermented orange peel and nano-selenium improved carcass weight and reduced abdominal fat weight through their antioxidant, metabolic, and gut health-enhancing effects. The fermentation process helps increase the bioavailability of these supplements and increase the secretion of enzymes, thus improving nutrient utilization (Predescu et al., 2024). Furthermore, it improves gut health, enhances protein and energy utilization efficiency, and reduces oxidative stress (Xu et al., 2023), which enhances muscle growth. It also improves the balance of gut microbiota and increases the production of beneficial short-chain fatty acids (Zhu et al., 2023), thereby improving digestion and absorption. Moreover, nano-selenium plays a crucial role in protecting cells from oxidative damage caused by heat stress by enhancing the activity of antioxidant enzymes, protecting muscle cells from oxidation and damage, and supporting thyroid function and metabolism (Chen et al., 2024). This improves nutrient utilization efficiency, resulting in increased muscle protein deposition and higher carcass weight (Elbaz et al., 2023). Several reports indicate the role of selenium and orange peel supplements in enhancing lean muscle mass (El-Aziz et al., 2025). Additionally, studies have shown that adding fermented feed and selenium has had effects on reducing abdominal fat in chickens (Liu et al., 2023; Wang et al., 2023; Abdelhady et al., 2025). Therefore, fermented orange peel and nano-selenium supplements can have an integration effect when used together, improving intestinal health and nutrient absorption, and reducing oxidative damage to muscle tissue, thus directing nutrients towards muscle building rather than fat deposition.

Increased oxidative stress, decreased feed consumption, damage to intestinal villi, and disruption of digestive enzyme activity during heat stress result in poor digestion and absorption of nutrients (Attia et al., 2024). However, in this study, the combination of fermented orange peel and nano-selenium enhanced the digestion of fiber and protein in heat-stressed chickens. The positive effect of adding selenium or fermented orange peel on the digestion and absorption of nutrients may be due to their role in reducing oxidative stress and intestinal inflammation (Elkhateeb et al., 2024; Predescu et al., 2024). Selenium protects the digestive tract from oxidative damage by reducing oxidative stress in intestinal cells, maintaining the integrity of intestinal villi, strengthening the intestinal barrier, and reducing intestinal inflammation (Elkhateeb et al., 2024; El-Aziz et al., 2025). Moreover, fermentation of feed using beneficial bacteria improves feed nutritional value through several mechanisms, including breaking down complex fibers, reducing antinutrients, increasing digestive enzyme production (Predescu et al., 2024; Alqhtani et al., 2026a), and supporting the production of SCFAs during fermentation, which improves intestinal cell health and provides an additional energy source (Xu et al., 2023). Additionally, orange peel contains many bioactive compounds with properties such as natural antioxidants, anti-inflammatories, and antimicrobials (Abd El Latif et al., 2023; Goliomytis et al., 2025), which improve fat and protein digestion and increase nutrient absorption. This is consistent with several reports that have stated that adding selenium supplements or orange peel supports nutrient digestion, in addition to the role of the fermentation process in promoting nutrient digestion and absorption.

In the current study, the addition of fermented orange peel and nano-selenium mixture enhanced the physiological processes in heat-stressed chickens by regulating lipid metabolism, lowering cholesterol and triglycerides, improving liver and kidney function, and enhancing stress index, thus helping to maintain vital performance. Selenium and orange peel act as powerful antioxidants (Zoidis et al., 2022; Liu et al., 2023) by reducing free radicals, which lowers lipid peroxidation, reduces fat accumulation in the liver, and protects liver and kidney cells from damage, which improving blood lipid profiles, lowers liver enzyme levels, and enhances kidney filtration efficiency (Abbasi et al., 2015; Abdel-Moneim et al., 2022). Additionally, fermentation improves physiological processes during heat stress by modifying gut microbiota and increasing the production of SCFA (Predescu et al., 2024; Wang et al., 2026), thus reducing inflammation, improving the health of vital tissues, and lowering the oxidative burden on the liver, kidneys, and thymus gland. Consequently, it enhances fat digestion and synthesis in the liver and improving metabolic energy balance (Zhu et al., 2023; Sızmaz et al., 2026), resulting in better blood lipid profiles and overall metabolism.

Heat stress in broilers leads to atrophy and weakening of immune organ activity, thus reducing the chickens' resistance to disease and impairing the efficiency of their immune response (Abdel-Moneim et al., 2021). Despite that, adding fermented orange peel and nano-selenium mixture enhanced the immune response in heat-stressed chickens, as evidenced by an increase in the relative weight of the bursa of Fabricius and levels of IgA, IgG, and IL-10, while serum IL-6 levels decreased. The increased relative weight of the bursa of Fabricius indicates improved growth and activity of B cells responsible for antibody production (Cheng et al., 2023), while elevated IgA and IgG levels suggest enhanced intestinal mucosal immunity and systemic immunity (Yu et al., 2021). Besides, elevated IL-10 and decreased IL-6 levels indicate a shift in the immune response towards an anti-inflammatory state (Ye et al., 2025), which helps heat-stressed chickens maintain their overall health. Selenium and orange peel are natural antioxidants and anti-inflammatories that reduce free radical formation and oxidative stress, protecting immune cells and intestinal cell integrity from oxidative damage (Zoidis et al., 2022; Goliomytis et al., 2025), especially during heat stress. Furthermore, fermented feeds produce SCFA, enzymes, and bioactive compounds that enhance the immune response (Alqhtani et al., 2026a). These enhance intestinal barrier integrity, stimulate mucosal immunity, and increase the availability of essential nutrients for immune cell formation which stimulates the growth and development of lymphoid organs such as the bursa of Fabricius. As well as, fermented feed raises IgA levels and promotes IL-10 production by improving microbial balance and reducing immune stress (Alqhtani et al., 2026a,b). Many reports have indicated the effective role of orange peel, selenium supplements, and fermented feeds in modulating the immune response (Goliomytis et al., 2025; Elbaz et al., 2026). Therefore, combining nano-selenium and fermented orange peel can immunomodulatory effect in broilers exposed to heat stress.

In the current study, the combined dietary supplementation of selenium and fermented orange peel enhanced oxidative stability in heat-stressed chickens by increasing the activity of oxidative enzymes (SOD, T-AOC, GPx, and CAT) and decreasing MDA levels. Selenium is one of the most important elements associated with antioxidant defense (Elkhateeb et al., 2024), through its role in the synthesis of many selenoproteins, such as glutathione peroxidase, which converts hydrogen peroxide and oxidized lipids into less harmful compounds (Attia et al., 2024). This, in turn, reduces the concentration of MDA and ROS (El-Aziz et al., 2024), thus minimizing damage to cell membranes. Additionally, orange peel contains high levels of bioactive compounds with a strong ability to neutralize free radicals (Zoidis et al., 2022), which increases the activity of antioxidant enzymes and reduces the accumulation of MDA an indicator of oxidative stress. They also help inhibit inflammatory pathways associated with the formation of ROS (Elkhateeb et al., 2024), thus improving overall oxidative balance. Besides, fermenting feed also improves its biological value by producing compounds including SCFA, vitamins, and microbial enzymes (Zhu et al., 2023), all of which possess antioxidant activity (Elbaz et al., 2023). Furthermore, it promotes beneficial microbes that activate the Nrf2 pathway (Wang et al., 2026), which regulates the gene expression of antioxidant enzymes (Xu et al., 2023; Abdelhady et al., 2025), thus enhancing oxidative stability within the body. Several previous reports have also indicated the positive effects of nano-selenium supplementation, orange peel, and fermented feed in promoting oxidative stability (Abdel-Moneim et al., 2022; Zoidis et al., 2022; Abdelhady et al., 2025). Therefore, combining nano-selenium and fermented orange peel can increase the efficiency of the body's oxidative defense system.

Supplementation with nano-selenium and fermented orange peel enhanced intestinal health by reducing pathogenic microbes, increasing SCFA levels, and decreasing acidity, in this study. Selenium helps maintain intestinal health, as an antioxidant, by reducing oxidative stress and inflammation within the intestinal mucosa and protecting epithelial cells and intestinal villi from oxidative damage (El-Aziz et al., 2025; Elbaz et al., 2026). Besides, orange peel contains fermentable fiber, pectin, flavonoids, and essential oils (Hussein et al., 2023; Abdelhady et al., 2025), which positively affect the intestinal environment by increasing fiber fermentation within the cecum and producing health-promoting SCFA (Han et al., 2023), and creating a favorable environment for the growth of beneficial bacteria (such as *Lactobacillus*) and inhibiting harmful bacteria. Additionally, feed fermentation is one of the most effective ways to improve intestinal health by reducing anti-nutritional factors and mycotoxins (Predescu et al., 2024), increasing the content of beneficial bacteria and organic acids in the feed, and lowering the intestinal pH due to increased production of SCFAs resulting from microbial fermentation activity (Elbaz et al., 2023; Zhu et al., 2023). SCFAs play a key role in strengthening the tight junctions between intestinal cells, nourishing intestinal cells (Liu et al., 2024), stimulating villus growth, reducing inflammation, and preventing pathogenic microbes from adhering to the intestinal wall (Liu et al., 2021). These properties align with numerous studies demonstrating the positive effects of nano-selenium, fermented feeds, and orange peel on modifying gut microbiota and increasing SCFA production, which promotes intestinal health (Fang et al., 2023; Abdelhady et al., 2025; Alqhtani et al., 2026b). Therefore, a dietary mixture of nano-selenium and fermented orange peel can enhance intestinal integrity in broiler chickens exposed to heat stress.

Consistent with our previous findings, adding mixture of fermented orange peels with nano-selenium increased regulation of gene expression of genes associated with intestinal integrity and nutrient transport, including MUC-2, SGLT-1, CLDN-1, ZO-1, and CAT-1, as well as increased expression of the IGF-1 gene. This suggests that adding the nano-selenium-fermented orange peel mixture improves intestinal function. Maintaining the integrity of the intestinal barrier is a key factor responsible for nutrient utilization efficiency and immune status in broiler chickens, especially under heat stress conditions (Abdel-Moneim et al., 2021). The increase in MUC-2 gene expression can be explained by the ability of supplements to enhance intestinal mucus secretion from goblet cells (Elbaz et al., 2025), which may be achieved by increasing the production of SCFAs, resulting from the activity of beneficial microbes that stimulate goblet cell differentiation and mucin secretion, which increases the thickness of the mucous layer, which represents the first line of defense against pathogens and enterotoxins (Alqhtani et al., 2026a,b). The improved gene expression of CLDN-1 and ZO-1 also reflects enhanced integrity of the junctions between intestinal cells, which limits the leaky gut phenomenon associated with heat stress (Duangnumsawang et al., 2023). This contributes to reduced intestinal permeability and prevents the transfer of endotoxins and inflammatory molecules into the bloodstream (Hossain et al., 2025), which may be due to the role of dietary supplements in modifying the gut microbiota responsible for this. On the other hand, increased gene expression of SGLT-1 indicates improved intestinal absorption of glucose and sodium from the intestinal lumen (Al-zaban et al., 2026), reflecting improved energy utilization efficiency. Furthermore, increased CAT-1 expression suggests improved transport and absorption of cationic amino acids (Alqhtani et al., 2026b; Elbaz et al., 2026), particularly arginine and lysine, which play a pivotal role in protein synthesis, tissue growth, and intestinal cell regeneration. Additionally, the results showed a significant increase in the gene expression of IGF-1, a key mediator of the growth hormone axis, which plays a major role in stimulating cell proliferation, protein synthesis, and muscle growth, suggesting an improvement in the physiological and metabolic status of the chickens (Abdel-Moneim et al., 2025). The increased IGF-1 expression associated with the nano-selenium and fermented orange peel mixture may be attributed to its antioxidant and antimicrobial properties. these gene-expression-modifying effects are likely due to the effects of dietary supplements via multiple physiological mechanisms, including improved gut microbiome balance, reduced inflammation, and reduced oxidative stress.

**In conclusion**, the results showed that a nutritional intervention based on a combination of fermented orange peel and selenium nanoparticles represents a promising nutritional strategy for improving the ability of chickens to adapt to heat stress conditions through integrated mechanisms, including enhanced antioxidant defense system and immune response, which contributed to improved growth performance. Furthermore, the dietary combination enhanced intestinal health, which demonstrated by modifying the balance of intestinal microbes, maintaining the integrity of the intestinal barrier, and enhancing gene expression associated with the digestion and absorption of nutrients, thus contributing to improved nutrient utilization efficiency. Therefore, incorporating fermented orange peel and selenium nanoparticles into feed may be an effective nutritional strategy to promote the health and performance of heat-stressed broilers.

## Funding

This research was funded by the Annual Funding track by the Deanship of Scientific Research, Vice Presidency for Graduate Studies and Scientific Research, King Faisal University, Saudi Arabia Project number (KFU264492). This research is also supported by Princess Nourah bint Abdulrahman University Researchers Supporting Project number (PNURSP2026R458), Princess Nourah bint Abdulrahman University, Riyadh, Saudi Arabia.

## Ethics declarations

Animal care procedures were conducted in accordance with the Ethical Committee of Animal Experiments of Desert Research Center, Cairo, Egypt, and King Faisal University, Saudi Arabia, which approved this study under protocol # KFU-REC-2026-MAY– ETHICS4205.

## Authors contribution

Ahmed M. Elbaz, Mohamed Marzok, Mohammed Al-Rasheed, and AbdelRahman Y. Abdelhady made substantial contributions to the conception and design of the study, conducted and/or supervised the research activities, contributed to the acquisition, analysis, and interpretation of the data, and participated in drafting the manuscript. Eman Kamel M. Khalfallah, Mohamed A. Sabry, Mayyadah Abdullah Alkuwayti, Haifa Ali Alqhtani, Mahmoud HA Mohamed, and Fatmah Ahmed Safhi made substantial contributions to the conduct of the study, data analysis and interpretation, and critically reviewed the manuscript for important intellectual content. Ahmed M. Elbaz, AbdelRahman Y. Abdelhady, Mohamed Marzok, and Haifa Ali Alqhtani contributed to the overall coordination and supervision of the research and provided the necessary research resources and support. All authors contributed substantially to the work, critically reviewed the manuscript, approved the exact final version for publication, and agree to be personally and publicly accountable for all aspects of the work, including ensuring that any questions related to the accuracy or integrity of the research are appropriately investigated and resolved.

## Disclosures

The authors declare no competing interests.
